# A new semi-automated workflow for chemical data retrieval and quality checking for modeling applications

**DOI:** 10.1186/s13321-018-0315-6

**Published:** 2018-12-10

**Authors:** Domenico Gadaleta, Anna Lombardo, Cosimo Toma, Emilio Benfenati

**Affiliations:** Laboratory of Environmental Chemistry and Toxicology, Department of Environmental Health Sciences, Istituto di Ricerche Farmacologiche Mario Negri IRCCS, Via la Masa 19, 20156 Milan, Italy

**Keywords:** QSAR, Data curation, Data cleaning, Semi-automated, Workflow

## Abstract

The quality of data used for QSAR model derivation is extremely important as it strongly affects the final robustness and predictive power of the model. Ambiguous or wrong structures need to be carefully checked, because they lead to errors in calculation of descriptors, hence leading to meaningless results. The increasing amounts of data, however, have often made it hard to check of very large databases manually. In the light of this, we designed and implemented a semi-automated workflow integrating structural data retrieval from several web-based databases, automated comparison of these data, chemical structure cleaning, selection and standardization of data into a consistent, ready-to-use format that can be employed for modeling. The workflow integrates best practices for data curation that have been suggested in the recent literature. The workflow has been implemented with the freely available KNIME software and is freely available to the cheminformatics community for improvement and application to a broad range of chemical datasets.

**Electronic supplementary material:**

The online version of this article (10.1186/s13321-018-0315-6) contains supplementary material, which is available to authorized users.

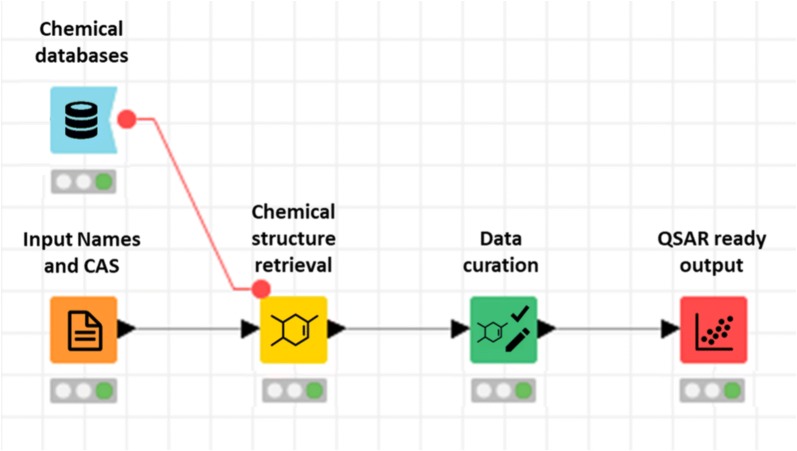

## Introduction

Quantitative Structure–Activity Relationships (QSARs) are statistical models relating a property/activity (i.e. endpoint) (e.g. pharmacological effect, or the toxicity, physico-chemical or bio-physical properties) of a set of chemicals to their structural features, encoded in a numerical notation by means of molecular descriptors.

**Fig. 1:**
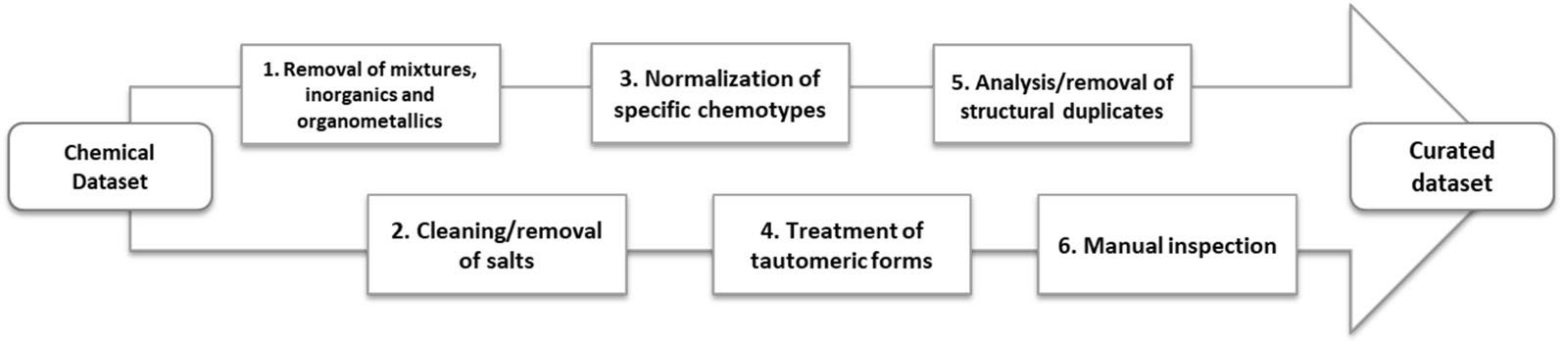
Workflow for data curation. Adapted from [2]

It is intuitive that a QSAR’s predictions cannot be more accurate than the original data used for its derivation [1]. Therefore, it is of the utmost importance that the dataset used for model derivation (i.e. the training set) contains high quality data, because any error in chemical structure or biological data will be implicitly transferred into the QSAR model. In these regards, a careful curation and selection of input data is essential [2] (Fig. 1).

With the advent of high-throughput technologies, increasing numbers of chemical data have been made available to the scientific community [3]. Consequently, more and more web-based data services and tools have emerged, that provide a way to store and constantly update information on thousands of different chemical structures. Examples include ChemIDplus [4] and PubChem [5].

The availability of these open access databases offers advantages in terms of chemical structure diversity but also disadvantages such as the lack of standardisation [6]. The lack of agreement on a unique identifier for chemical structures sometimes makes it difficult to compare and integrate structural data from different sources. The International Union of Pure and Applied Chemistry (IUPAC) international chemical identifier (InChI) code [7] has been proposed as a unique structural format to identify and compare chemical structures, e.g. checking for duplicates [8]. This kind of notation is nowadays widely accepted and used more and more. However, in lots of cases ambiguous identifiers such as the Simplified Molecular Input Line Entry System (SMILES), chemical names and chemical abstracts service (CAS) registration numbers are still used, especially in older datasets.

Another problem related to the increasing mass of data is often the lack of consistent structural and/or biological information on the same compound retrieved from different sources. It has been reported [9–11] that a substantial percentage not ignorable of structures in public and commercial databases contain errors.

For these reasons, more and more papers in the last years have highlighted the importance of a careful inspection and curation of chemical structures to derive QSAR models [2, 11–14]. The inclusion of ambiguous or wrong structures will lead to errors in the calculation of descriptors [15] and, consequently, to meaningless QSARs [14].

Manual inspection of large chemical datasets is impossible in the majority of cases, because they often include hundreds or even thousands of different chemical structures. The use of automated or semi-automated methods for data retrieval, curation and standardization is highly advisable [13].

At present, however, only few automated methods are available to the scientific community, most of them addressing only some of the above-mentioned aspects (data retrieval, curation, standardization).

The literature still lacks a unified and organized protocol to prepare and curate compound data for QSAR modeling, even though this need has been highlighted several times [2, 13]. Here we present a new semi-automated procedure to support scientists in data preparation for modeling purposes. The procedure addresses all three steps of (1) data retrieval, (2) data curation and (3) standardization of chemical structures. Data (i.e., SMILES) was automatically retrieved from different, orthogonal web-based databases, using two widely used identifiers, i.e. chemical name and CAS registration number. The use of non-redundant sources is essential because ensures an unbiased comparison of retrieved information [13].

Records were scored based on the coherence of information retrieved from different web sources. Top scored records were then curated. This includes removal of inorganic and organometallic compounds and mixtures, neutralization of salts (but maintaining the information about the counterions in a separate attribute), removal of duplicates (and checking for tautomeric forms). Finally, the resulting SMILES are converted to a standardized format, yielding ready-to-use data for the development of QSARs.

The entire workflow was implemented in KNIME (version 3.4) [16] and made freely available to the cheminformatic community to use and improve. The workflow is dedicated to the structural checking of data, making it suitable for application to different types of chemical datasets, regardless of the endpoint considered.

## Materials and methods

### Description of the database

To asses the efficiency of the workflow, it was applied to three datasets available in the literature, covering different chemical spaces. The first dataset was compiled by Obach et al. [17] and includes 670 drugs together with experimental intravenous data for some relevant pharmacokinetic parameters. For each compound two identifiers, i.e. chemical name and CAS number, were provided and used as input for the KNIME workflow.

The second dataset was the SIN-list compiled by ChemSec [18]. It includes data for 913 industrial chemicals identified by ChemSec as substances of very high concern, based on the criteria for these defined by article 57 of REACH (EC [19]). These include carcinogens, mutagens and repro-toxic substances; persistent, bio-accumulative and toxic substances; substances of “equivalent concern” of these two categories (e.g. endocrine disruptors).

The third dataset was the EPISuite™ water solubility dataset. It includes 5761 compounds with CAS number and chemical name. This dataset was used by US EPA to develop the WSKOWWIN v 1.42 model to estimate the water solubility (https://www.epa.gov/tsca-screening-tools/epi-suitetm-estimation-program-interface). For chemicals included in the three datasets, CAS numbers and chemical names were used for searching SMILES. Eleven substances in the SIN List were identified by more than one CAS number, and only the first was considered for searching SMILES.

### Description of the workflow

**Fig. 2:**
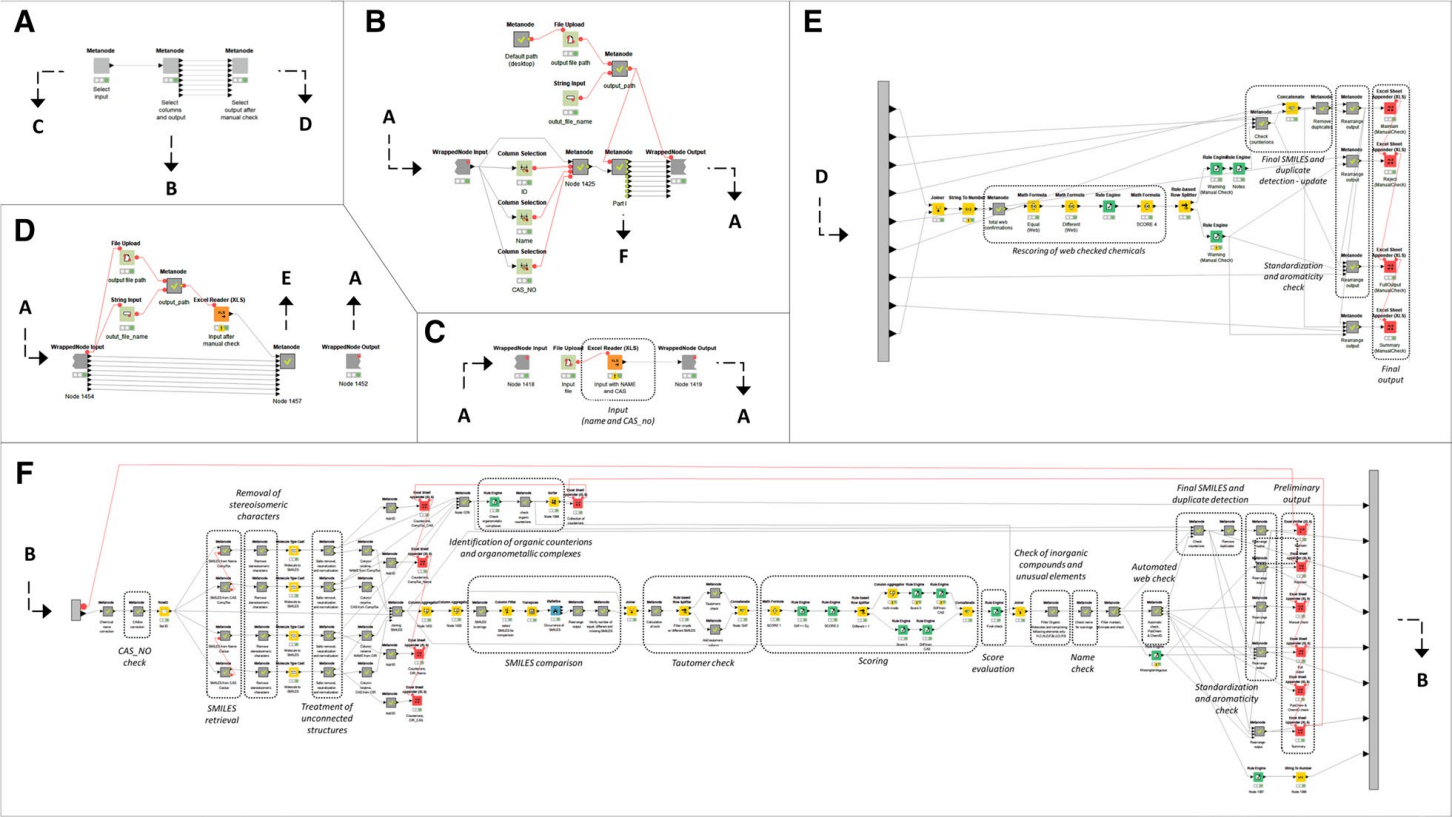
Representation of the KNIME workflow for data retrieval and curation

The entire procedure described below was implemented as a KNIME workflow (https://www.knime.com/) [16], which is freely available for download at https://github.com/DGadaleta88/data_curation_workflow. A guidance for users explaining how to install and use the workflow is also provided at the same link and in the Additional file 1. The workflow is depicted in Fig. 2.

#### Data retrieval

The workflow can retrieve SMILES using both the chemical name and the CAS registration number. The workflow accepts input data from a user supplied tabulated file, with columns including the lists of the two identifiers. CAS numbers are preliminarily checked for the presence of leading zeros and unexpected characters. Zeros are removed, while anomalous characters are flagged with a warning. A checksum is also performed on CAS numbers to verify their correctness [20].

Chemical names and normalized CAS numbers are converted into fixed URLs that are sent to a Representational State Transfer (REST) service. The REST service will automatically make queries to a web service connected to publicly available chemical databases, resolving chemical identifiers to SMILES. In the present workflow, four services are used:

- Chemical Identifier Resolver (CIR) from CADD Group Chemoinformatics Tools and User Services (CACTUS) (https://cactus.nci.nih.gov/chemical/structure) provided by the National Cancer Institute (NCI) [21];
- CompTox Chemistry Dashboard [22] (https://comptox.epa.gov/dashboard) from the U.S. Environmental Protection Agency (EPA) integrating DSSTox (https://www.epa.gov/chemical-research/distributed-structure-searchable-toxicity-dsstox-database), ACToR, ToxCast (https://www.epa.gov/chemical-research/toxicity-forecasting), EDSP21 (https://actor.epa.gov/edsp21/) and CPCat (https://actor.epa.gov/cpcat/faces/home.xhtml) chemistry resources;
- PubChem (https://pubchem.ncbi.nlm.nih.gov/#) [4];
- ChemIDPlus (https://chem.nlm.nih.gov/chemidplus/) provided by the National Institute of Health (NIH) [5].

CIR and CompTox services are employed in a first run of the workflow to retrieve SMILES for all the input chemicals using names and CAS numbers as queries. In the second part of the workflow, records showing incongruent information from the first two databases are checked on PubChem and ChemIDPlus, as explained in “Manual check” section.

#### Structural data cleaning

In the first part of the workflow up to four SMILES are recovered for each record from two chemical databases (CIR and CompTox) using chemical names and CAS numbers as identifiers. A preliminary step to raise the quality of retrieved data consists in identifying and removing the fraction of data that cannot be appropriately handled by the majority of modeling softwares, such as inorganic and organometallic compounds, isomeric mixtures and data related to mixtures of chemicals. The remaining structures need to be treated appropriately, for example salts are neutralized and counterions eliminated [2, 13]. In this case, the information about the presence of a salt and the counterions eliminated are maintained as a separate attribute (as described below). This means that the user can retrieve this information when necessary.

All these operations are done in the workflow using publicly available nodes from the Chemical Developmental Kit (CDK) (https://cdk.github.io/) (version 1.5.600) and RDKit (http://www.rdkit.org/) (version 3.2.3) toolkits, together with standard KNIME cheminformatics nodes.

In case of unconnected structures, such as salts, counterions are separated from the main molecule (identified as the one with the highest molecular weight) with the CDK connectivity node. After this, the main molecule is converted to the neutral form when possible, using the RDKit Structure Normalizer node. A flag is added to indicate when neutralization of chemicals was successful, or when errors arose in the neutralization. Another flag indicates the presence of metallic or organic counterions. These operations are done on all the SMILES retrieved from the various web sources. The information related to the original SMILES, the neutralized form and each counterion are stored in the workflow.

Characters encoding stereoisomerism (@; \; /) are removed because this information is usually not relevant for classical QSAR derivation (that works in 2D). Chemicals undergoing this operation are flagged accordingly.

SMILES are checked for the presence of tautomers. This is done to avoid apparent inconsistency based only on differences in the tautomeric form of analogous chemicals [23]. InChI [24] are derived from each SMILES using the RDKit to InChI node and employed to check tautomers. InChI is a unique identifier that allows to univocally identifies a given chemical. In the same way, identical substances always receive the same label (under the same labeling conditions). This is achieved through a well-defined procedure of canonical numbering of atoms.

An InChI is made of a series of layers, i.e. character sequences starting with a forward slash. Each layer encodes specific information for a given chemical, such as the empirical formula, stereochemistry and isotopic mass of atoms. The last layer lists the exact position of tautomeric hydrogens [24]. Two stereoisomeric forms of the same chemicals share the same InChI, except for the last layer. For this reason, the first layers of InchI were used to compare different SMILES for a given record. Inconsistencies between SMILES generating InChI codes analogous for the first layers are results of different tautomeric representations and are considered equal in the subsequent steps of the procedure.

In the second part of the workflow compounds that passed the selection procedure (see “Data scoring and selection”) are checked for the presence of unusual elements (i.e. those different from H, C, N, O, F, Br, I, Cl, P, S) with an element filter node by CDK extension. Those, together with inorganic compounds, are removed from the final list of chemicals.

Chemical names are checked for keywords flagging for not univocal or problematic structures (metabolites, formulations, mixtures, degradates, reaction masses/products, products, isomers, polymers, derivatives, chemical substances of unknown or variable composition, complex reaction products and biological materials (UVCBs)) and flagged accordingly. Mixtures, reaction masses/products, UVCBs and polymers are removed because the relative endpoint values often refer to multiple chemicals that should not be considered for modeling purposes. The remaining cases are moved to a list of chemicals needing manual checking, while isomeric mixtures are kept because stereoisomery has already been stripped in the first steps of the procedure (see above).

#### Data scoring and selection

The equal (i.e., consistent) (E), different (i.e., incongruent) (D) and missing (M) SMILES are counted for each record. Comparison is made on the first layers of InChI codes directly generated from the retrieved SMILES. InChI are particularly suitable to perform this comparison because, unlike SMILES, they identify a structure univocally, also avoiding inconsistencies caused by different tautomeric representation of the same compound. These counts are used to compute a score for each record. Based on the level of incongruence among SMILES, records with lower scores are rejected or require manual checking.

The score is calculated based on Eq. (1):

1 $$Score = E - \left( {D \cdot 0.2} \right)$$

If D is greater than E for a record, the score is forced to zero. The final score ranged from four (all the SMILES are congruent) to zero. In addition, the score is forced to zero when the two SMILES from the CAS lookup (from CIR and CompTox) are not equal. The CAS number is usually a more reliable identifier than the chemical name, because it is less affected by typos and syntax errors. This means, incongruencies in SMILES retrieved from CAS are more serious than those in SMILES from chemical names. A high final score represents a proof-of-evidence of the real match existing between the retrieved structure and the identifiers provided as input.

Based on this final score, records are divided into Maintain (Ma), Check (C) and Reject (R).

*Ma chemicals* (Score ≥ 3). These have enough concordant information to be included in the final curated list of chemicals. Records with Score = 4 (four equal SMILES) have maximum reliability, those with Score = 3 (three equal, one missing SMILES) have medium reliability.

*R chemicals* (Score = 0). These have highly discordant or totally missing information, or belong to class of compounds that typically are not considered for modeling (e.g. inorganic, mixtures, complexes. See “Structural data cleaning” section). These compounds are no longer considered for inclusion in the final dataset.

*C chemicals* (Score < 3) require further assessment based on the source of the missing/incongruent information:

- SMILES from Name are missing both from CIR and CompTox. In this case, the name reported in the input may contain some typos, or it is an unused synonym. The consistence of the chemical name must be checked and at least another confirmation of SMILES obtained from the chemical name should be searched in other databases.
- Only one SMILES is retrieved and the other three are missing. In this case, at least two more concordant SMILES are required from two new different databases.
- Two SMILES are concordant but two are missing, or three SMILES are concordant but one is different. In this case, at least one confirmation should be found from other databases.

#### Manual check

C records needing only a check of the chemical name were automatically verified on PubChem REST Service (PUG) (https://pubchemdocs.ncbi.nlm.nih.gov/pug-rest), as described at “Data retrieval”. The CAS number was used as input to retrieve all possible synonyms for the chemical included in PubChem. If the input chemical name matches one of the synonyms in the list, the consistency between Name and CAS is confirmed and the record is moved from C to Ma. Otherwise, the name must be further checked manually. The list of synonyms retrieved from PubChem is maintained in the preliminary input document generated by the workflow, in order to facilitate visual inspection of names.

For C records requiring one or two confirmations of the SMILES retrieved in CIR or CompTox, automated retrieval of SMILES is done on PubChem and ChemID starting from both CAS number and Name. The entire procedure already performed for SMILES retrieved from CompTox and CIR is repeated (removal of counterions, neutralization, check for tautomers, normalization of SMILES, scoring based on the count of concordant evidence). Retrieved structures are compared with those retrieved by CompTox and/or CACTUS. If enough confirmations are collected, the record is moved to M chemicals.

Chemicals that fail the automated check need to be verified manually. The workflow produces a preliminary output document listing the chemicals for which it was not possible to find confirmation and that are likely to be excluded. Possible sources for the manual web check should be different from the databases used in the procedure described (e.g., ChEMBL [25], ZINC [26], ChemSpider [27], DrugBank [28]), supplier/distributor websites, public dossiers from agencies or structure repositories for specific chemicals (e.g. repositories of drug compounds).

An empty field in the preliminary document should be filled by the user with the number of new confirmations found. Then the updated document is loaded again in the workflow for the last part of the procedure. Checked chemicals are rescored based on the number of new confirmations. If there are enough confirmations, chemicals are moved to Ma, otherwise they are rejected.

**Fig. 3:**
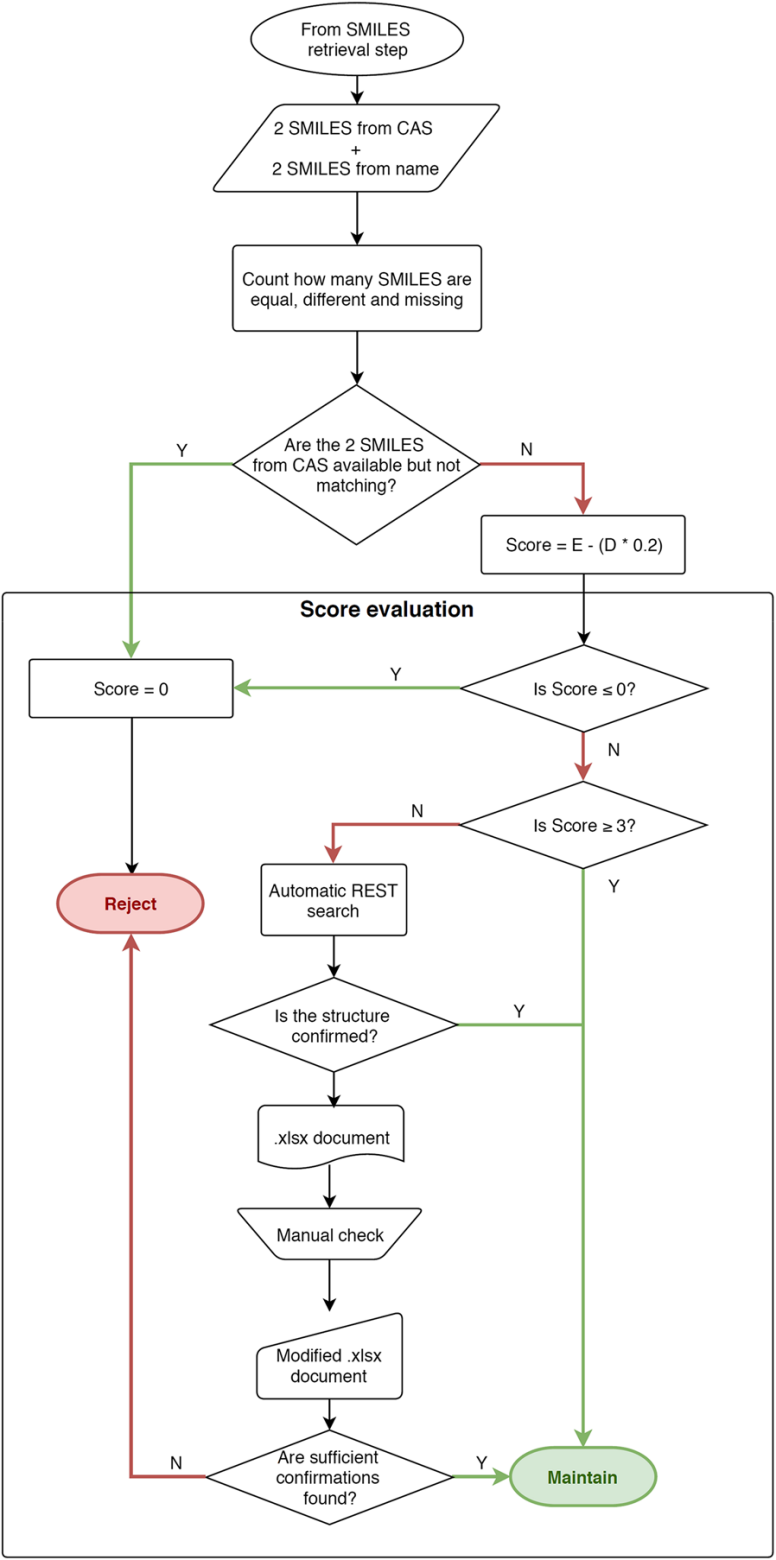
Workflow describing the scoring scheme of chemicals in the data curation procedure. *E* equal, *D* different

Table 1 reports the possible combinations of congruent, incongruent and missing structures from the four different combinations of sources and identifiers (Name/CIR, Name/CompTox, CAS/CIR and CAS/CompTox), the score assigned and the any manual check required. Figure 3 shows the entire scoring scheme.

**Table 1:** Case series and corresponding scores

E	D	M	Note	Score	Modified score	Check	Manual check notes
4	0	0		4.0	4.0	Ma	
3	0	1		3.0	3.0	Ma	
3	1	0	CAS_CIR = CAS_CompTox	2.8	2.8	W	Verify name
3	1	0	CAS_CIR ≠ CAS_CompTox	2.8	0.0	R	
2	0	2	SMILES from name are both missing	2.0	2.0	C	Verify name
2	0	2	The two SMILES from one source are both missing	2.0	2.0	C	Search for at least one confirmation
2	0	2	One SMILES from CAS and one SMILES from name are missing from different sources	2.0	2.0	C	Search for at least one confirmation
2	0	2	SMILES from CAS are both missing	2.0	2.0	C	Verify correctness of CAS; search for at least one confirmation
2	1	1	CAS_CIR = CAS_CompTox	1.8	1.8	C	Verify name
2	1	1	CAS_CIR ≠ CAS_CompTox	1.8	0.0	E	
1	0	3		1.0	1.0	C	Search for at least two confirmations
0	0	4		0.0	0.0	R	
2	2	0		1.6	0.0	R	
0	2	2		− 0.4	0.0	R	
0	3	1		− 0.6	0.0	R	
0	4	0		− 0.8	0.0	R	

For each possible combination of equal (E), different (D) and missing (M) SMILES, the table report the assigned score, the final check [maintain (Ma), reject (R) and manual check (C)] and instruction for checking C chemicals

#### Structural normalization

The final list of neutralized SMILES is converted into a standardized QSAR-ready format. The OpenBabel KNIME implementation [29] is used to generate Canonical SMILES [30]. In addition, a kekulizer node from RDKit extension is used to solve ambiguous aromatic structures and generate SMILES with explicit aromaticity. Counterions of SMILES from different sources are compared, and differences are flagged. At this stage duplicates in the list are detected and removed with a check on the first three layers of InChI generated from SMILES.

#### Output

The workflow produces an *.xlsx document comprising various sheets, each giving different information:

- *Maintained:* this includes (i) a new progressive ID for chemicals; (ii) all the original SMILES; (iii) the neutralized SMILES with explicit aromaticity; (iv) the Open Babel neutralized Canonical SMILES; (v) the number of duplicates for the record in the original dataset; (vi) the list of names (with occurrences) assigned to the original records; (vi) the list of CAS_NOs (with occurrences) assigned to the original records; (vii) reliability (High or Medium); (viii) list of warnings.
- *Rejected:* this includes (i) ID; (ii) name; (iii) CAS_NO; (iv) a collection of warnings indicating the reason for removal of the record.
- *Manual Check:* list of compounds to be manually searched on the web. This includes (i) ID; (ii) name; (iii) CAS_NO; (iv) neutralized SMILES collected from CIR and CompTox; (v) number of Equal, Different and Missing SMILES; (vi) a specification of the information that must be searched on the web (verify consistency of the name, search for one or two further confirmations); (vii) a list of synonyms retrieved from PubChem for a preliminary name verification; (viii) an empty field to fill with the number of confirmations.
- *Full output:* this summarizes the information reported above for all the chemicals.
- *Neutralized and Counterions:* this includes (i) ID; (ii) CAS_NO; (iii) original SMILES; (iv) neutralized SMILES; (v) counterions retrieved from CIR and CompTox using Name and CAS_NO as identifier; (vi) warnings on counterions.
- *PubChem_ChemID_check:* this includes the same information as above, that results from the automated check on PubChem and ChemID.
- *Summary:* this reports the number of Maintained (including and excluding duplicates, with details on reliability), web check and rejected chemicals.
- *Counterions_*CompTox*/CIR_CAS/Name*: four parts that report: (i) original SMILES; (ii) SMILES stripped of counterions; (iii) neutralized SMILES; (iv) list of counterions; (v) MW of molecule and counterions; (vi) possible warnings for neutralization.

After the user check, the .xlsx file is reloaded in the workflow to include in the final output all the chemicals passing manual inspection. A series of new sheets are added to the final output, reporting the same information as above but on the entire dataset.

An example of output generated by the workflow for Obach dataset,  SIN List from ChemSec and EPISuite™ solubility dataset analysed in this article (see “Description of the database”) are reported in the Additional file 2, Additional file 3, Additional file 4.

## Results and discussion

We presented a new semi-automated procedure to retrieve structural data (SMILES) using different chemical identifiers as input (CAS number and chemical names). However, this is not a simple retrieving procedure, as a series of steps for structural data curation have also been implemented. The final output of the workflow is a curated QSAR-ready dataset comprising only reliable and high-quality data that can be used for modeling exercises. The workflow also integrates steps aimed at identifying errors and incongruences in the data. Because it is often impossible to check very large datasets manually because of prohibitive time requirements, this check has been partially automated in the workflow.

One example of quality checking is the verification of the consistence of chemical structures. This can be done automatically by retrieving and comparing structures from different publicly available datasets. Several authors [9, 11] have highlighted the presence of random human errors committed during compilation of public datasets. Retrieval of information from several sources is an easy way to recognize these errors. Using of the same chemical identifier (e.g. CAS, chemical name) to mine different datasets should lead to the same chemical structure. If the same structure is always retrieved using different identifiers from several, orthogonal databases, it is highly plausible that a real match exist between the final structure, the CAS number and the chemical name, and that no errors occurred in the source databases. Different results should be interpreted as an error in one or more of the datasets queried.

This automated identification of errors greatly improves the quality of the starting data. Quality is further gradually increased through various levels of structural data curation. These involve the removal of a part of the data that cannot be appropriately handled by the majority of modeling softwares, such as inorganic, organometallic compounds and mixtures of chemicals. The neutralization of ionized structures and the elimination of counterions are other commonly applied procedures [2] that have been implemented in the workflow.

In the last part of the curation procedure, the workflow provides a form of standardization of chemical structures in order to make them ready to use in modelling. Standardization of the structures is of the utmost importance because it has direct consequences on computed chemical descriptors. If two chemicals are similar, but are standardized in different ways, values for some descriptors may be different, resulting in errors in the model [14]. A classic example is the nitro group, which can be represented in several ways. In this specific case, descriptors based on the count of this particular functional group may lead to different results depending on the graphical representation of the group [13, 14]. Tautomeric forms [23] are another common issue that justifies the need for a rigorous standardization process. The selection of one form instead of another leads to modifications in connectivities and hydrogen positions that may produce differences in some of the molecular descriptors based on these properties.

Another common error caused by the lack of standardization is the presence of replicate structures [8]. This is mainly due to the fact that a same compound can be characterized by different notations or different structural representations (e.g., the same compound can be represented by different SMILES notations).

**Fig. 4:**
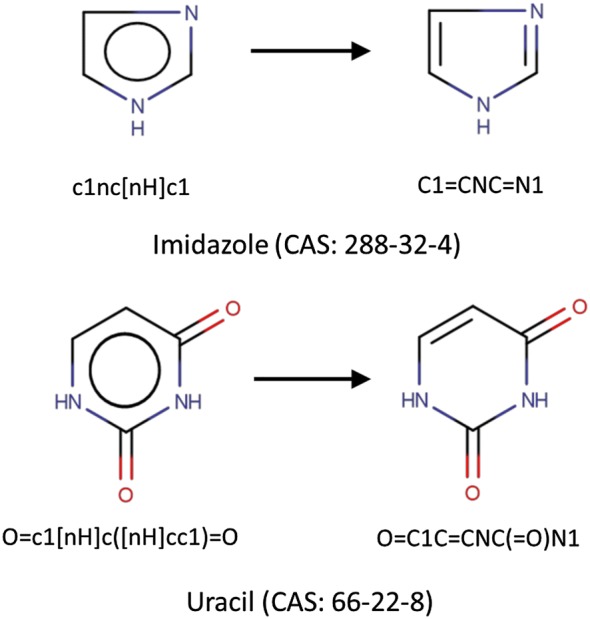
Explication of aromaticity for some problematic chemotypes

Aromaticity also needs standardization, particularly for some problematic structures, such as five-term aromatic compounds or aromatic rings with branched keto-groups. Public datasets, including those considered in the workflow, often include misleading representations of chemicals, including chemotypes that lead to errors in the calculation of descriptors related to the number of aromatic atoms and/or aromatic bonds in the molecule. The implementation of a kekulizer node in the workflow can handle this kind of issue. The node can explicate the aromaticity of problematic chemotypes, and univocally represent some atoms that are often wrongly reported as aromatic in SMILES, on account of these misleading representations (Fig. 4).

The authors intentionally defined the procedure as “semi-automated”, because there are some errors that are obvious to a human, but are still not obvious for computers. A final manual intervention is required to check the presence of errors that cannot be identified by a completely automated procedure. These included, for example, typos in chemical names or CAS number.

In order to guide the user in identifying errors that went unnoticed in the automated workflow, a series of notes and warnings are produced at each step of the curation procedure. Some of them are useful to drawn attention to steps that are most likely to generate errors. An example is the inclusion of flags for the presence of organic counterions. Sometimes it is impossible to determine the real role of a counterion in the biological activity of a compound. This is particularly true in the case of organic counterions with molecular weight similar to the main molecule. Here, the record is possibly a mixture of several ingredients, and manual deletion of the record is recommended, unless the user knows which component is responsible for the biological activity [13]. Sometimes a counterion is equal to the main molecule. In this case, the record is a dimer, and this should be taken into consideration when evaluating the experimental value for the record (i.e. influence of the stoichiometry of the record on the endpoint value).

In addition, in some cases neutralization of the salts is not appropriate for the purpose (e.g. for developing models for water solubility). For these reasons, the information about the counterions eliminated is reported in a dedicated sheet. The user can manually verify whether the counterion is organic or inorganic, whether it is mono or polyatomic, and whether all the structures retrieved for the same compound contain the same counterion (e.g. sodium and potassium salts, or sodium salt and acidic form).

The main limitation in formalizing a standard data curation procedure is the absence of a single tool able to handle both data retrieval and structural data curation. In house scripts (e.g. Python, R, Pearl) are useful for accessing public databases for SMILES retrieval, but the majority are limited to internal laboratory use and are not always available. They also often require proficiency with the programming languages to be used. For data curation, there are some software programs such as ChemAxon [31] Tool, MOE [32] and OpenBabel [29], implementing functionalities for counterion removal, neutralization of salts, removal of duplicates and standardization of chemical structures. However, almost no tools integrate both the data retrieval and curation procedures. The workflow presented here is one of the first attempts to provide a comprehensive automated tool to assist scientists in the preliminary steps to QSAR modeling.

One of the few and most notable example of automated curation of data proposed in the past is the procedure recently published by Mansouri et al. [33]. The authors introduced an automated workflow for the curation and correction of errors in the structure of chemicals. It compares structural information retrieved from four identifiers (chemical name, CAS number, SMILES and MolBlock) and the identification of mismatches and incongruences among these data. Stereoisomerism and different tautomeric forms are also addressed during the comparison. Last, a score was assigned to each data point based on the degree of consistency among the four identifiers. The procedure can be used in sequence with a second KNIME workflow implementing a series of rules for chemical data curation, such as removal of counterions, neutralization, removal of inorganic compounds, and standardization of tautomer representation [34].

This procedure has the limitation that it considered only one source (the DSSTox database from EPA) (https://www.epa.gov/chemical-research/distributed-structure-searchable-toxicity-dsstox-database) [35] for data retrieval, and in very few cases MolBlock is available.

One of the notable features of the data curation workflow is the assessment of reliability for each record maintained in the final list. This reliability may be “medium” or “high”. It reflects the score assigned to the record during the cleaning procedure and consequently the level of consistency among SMILES retrieved from the different data sources. This is extremely important because it enables the user to compile datasets with different levels of reliability and data quality, based on the needs and the number of compounds available. For example, if the curation procedure retains a large number of chemicals, one can choose to retain only entries with optimal quality that are safest for QSAR modeling. This is the ideal situation. However, in case of a dataset with quality and/or consistency issues, or only a moderate number of maintained records, the user may decide to keep both high and medium reliability records to obtain a sufficiently populated dataset for modeling.

The percentage of high and medium reliability records returned from the curation procedure also gives the user an overall view of the quality of the starting data. In the same way, the percentage of chemicals rejected by the procedure or needing a manual check give clues to the quality of input data. In other words, with datasets including chemicals that have a unique, well-known structure, such as drug chemicals, we may expect a very small number of rejected or dubious compounds, plausibly related to errors in CAS number or chemical names. On the other hand, running the workflow on datasets of industrial chemicals will very likely lead to a larger number of rejected chemicals and ambiguous results. It is common in this kind of dataset to find problematic entries such as mixtures, reaction products, polymers, etc. that should be excluded from modeling.

Table 2 summarizes the data curation procedure on the Obach dataset, the SIN list from ChemSec and the EPISuite™ dataset. The first two datasets illustrate opposite cases of data quality and were treated with the semi-automated procedure, in order to show differences in cleaning results. The third dataset represent a larger collection of data with respect of the first two, and was selected to show the suitability of the procedure on larger datasets and its relevance in terms of time save.

**Table 2:** Data cleaning results on the dataset from Obach et al. [17], the SIN list from ChemSec [18] and the EPISuite™ solubility dataset

Category	Obach et al. [17]	SIN list [18]	EPISuite™
Total	668	913	5761
Maintain (w/duplicates)	607	256	4635
Maintain (w/o duplicates) (H reliability)	514	163	3536
Maintain (w/o duplicates) (M reliability)	85	68	850
Manual check	47	115	639
Rejected (mixtures)	0	23	9
Rejected (inorganic or unusual elements)	2	194	96
Rejected (missing/ambiguous)	12	394	395
Maintain (manual check) (w/duplicates)	652	335	5014
Maintain (manual check) (w/o duplicates) (H reliability)	515	163	3554
Maintain (manual check) (w/o duplicates) (M reliability)	128	127	1171
Rejected (manual check failed)	2	36	260

Numbers refer to results before and after the manual check procedure

The first dataset includes a series of Adsorption, Distribution, Metabolism, Excretion (ADMET) properties for 668 drug compounds [17]. In recent years computational methods have proved particularly adequate for the prediction of pharmacokinetics and/or ADMET properties [36], making it extremely important to compile curated datasets for the derivation of QSARs predictive of these endpoints. For drug compounds it is often easy to retrieve consistent information as the drug structures are usually well documented and ascribable to a single chemical structure.

Out of the total of 668 records, 607 (91% of the initial dataset) passed the cleaning procedure after the first curation step. Eight pairs of duplicates were found, mostly due to the removal of stereoisomerism (e.g., levofloxacin and ofloxacin; betamethasone and dexamethasone); 514 single structures (about 86% of the chemicals maintained) were considered highly reliable, and 85 were flagged for minor reliability issues. Only 14 records (0.02% of the initial dataset) were rejected, one being inorganic (carboplatin), two having unusual elements (bortezomib and carboplatin) while 12 presented major reliability issues. As expected, no mixtures or other kinds of problematic chemicals were found in this dataset. A manual check was required for 47 records. The manual check of these chemicals is not hard because, as already specified, the structures of the drugs are in the majority of cases well known and documented and they can be easily retrieved from different public sources. This make it easy to identify possible inconsistencies due to errors and consequently to recover the majority of these chemicals. We managed to retrieve 24 of them simply by comparing the name with the synonym automatically retrieved from PubChem, while 19 of the remaining 23 were recovered after manual examination. Only two records were rejected, one because it was a metal complex (gadoversetamide) and one because it showed conflicting information from web sources consulted. Chemicals maintained were further inspected for the presence of organic counterions. In the end, 652 chemicals corresponding to 643 single records were kept from the original 668 records. This very high percentage of maintained chemicals was expected, and manual inspection was needed only for 47 records out of 668. In this specific case the usefulness of an automated procedure is clear in terms of time saved.

The SIN list (http://sinlist.chemsec.org/) from ChemSec shows a different picture with respect of the Obach dataset. It is made up of a more heterogeneous collection of industrial chemicals including almost all kinds of problematic cases in terms of data set curation: mixtures, inorganic compounds and entries with consistency and quality issues. On a total of 913 records, only 256 (28% of the initial dataset) passed the first run of the cleaning procedure, and 542 entries (59% of the whole dataset) were immediately rejected. For 394 of the rejected structures, retrieved SMILES presented problems of ambiguity or were totally missing. This is not unexpected, indeed it is common to find multiple industrial chemicals registered under a single CAS number (e.g., mixtures of structural isomers, mixtures of hydrocarbons with different lengths and degrees of branching), making it impossible to assign a single structure to these entries.

Of the total number of rejected chemicals, 194 were inorganic or included unusual elements, while 23 were mixtures. As explained, the real number of mixtures is probably higher if we consider those probably included in the large number of ambiguous or missing entries. A manual check was required for 115 chemicals. While in the Obach dataset only two chemicals did not pass the manual examination, in this case 36 out of 115 chemicals were rejected. Among those entries, mixtures of glycols or hydrocarbons (e.g. paraffin) with different lengths were found and removed because they have not got a fixed structure. Mixtures of stereoisomers were retained because stereochemistry was ignored. It was possible to recover some records showing typos or having more synonyms in the name field, separated by colons or semicolons. Ten chemicals were recognized as metal complexes. In the end, the manual check procedure retrieved a total of 335 records, i.e. 37% of the initial compounds. This is far from the 91% of retrieved entries of the Obach dataset, giving a clear indication of the different quality of the two datasets. The same sort of conclusions can be drawn from the percentage of high-reliability chemicals, which is 56% (163 out of 290 single maintained structures), much lower than the 80% of Obach data. After a further check of maintained structures we identified and removed 13 additional metal complexes (seven single structures, four with high and three with medium reliability) that erroneously passed the cleaning procedure. Such structures are often not recognized by the automated procedure, but they can be easily spotted in a second manual inspection among chemicals flagged with “Organometallic” and “Organic counterion” warnings. In this case, the organic counterion is the same main structure that is repeated several times in the complex. This is a clear demonstration of the importance of a manual inspection of the final results, especially with such problematic datasets.

The workflow was also run to a third dataset, to demonstrate the effectiveness of the tool on a larger set of data. The EPISuite™ water solubility dataset includes a total of 5761 chemicals (https://www.epa.gov/tsca-screening-tools/epi-suitetm-estimation-program-interface). After the first run of the cleaning procedure, 4635 records (80% of the initial dataset) were kept, corresponding to 4386 unique structures (3536 with high reliability and 850 with medium reliability). A total of 487 records (about the 8% of the initial dataset) were immediately rejected. The majority of them (395) showed missing or ambiguous information retrieved from CAS and chemical names, while 96 were rejected because they were inorganic or they include unusual elements. Nine records were recognized as mixtures. As expected, these percentages were in the middle of the values observed for the previous datasets, that were specially selected because they represent two opposite, extreme cases. A manual check was required for 639 out of 5761 records. 379 records passed the manual check procedure, while 260 were rejected. Several samples were rejected because the chemical name reported in the dataset was truncated, and was impossible to retrieve a SMILES from it. In some cases the manual check identified wrong SMILES that were initially retrieved from the CompTox or the CIR datasets (e.g., CAS 118247-04-4, 116482-80-5 or 116482-75-8, see Additional file 4) or identify incongruences between name and CAS (e.g., 3-ethyl-piperidine was wrongly associated in the dataset to the CAS 1484-80-6, that is the 2-ethyl analogue). As observed for the previous datasets, the manual check also allows to identify some typos that prevent to retrieve SMILES. In the end, 5014 records corresponding to 4725 single chemicals were kept from the original 5761 records. The number of chemicals that needed to be checked by the user were definitely more than those in the previous datasets. This is a consequence of the higher number of records in this dataset. Despite this, the automated procedure allowed to sensibly reduce the manual intervention made by the user that had to make a check on about the 10% of the initial records. This clearly highlight the importance of the automated procedure in terms of time save and its suitability for screening large collections of data.

Information on the starting quality of a dataset is extremely important and should always be kept in consideration for a critical evaluation of the results of modeling exercises. QSAR models derived from datasets characterized by a great percentage of data with moderate reliability cannot be expected to give results of the same quality as models based only on high-quality data. This is all the more evident with larger portions of moderate-quality data.

Currently, the workflow here presented is tailored for chemical structure cleaning, and it does not address endpoint data curation.

Endpoint curation, however, is another important aspect that should be considered when preparing a dataset for QSAR modeling because QSAR predictivity will reflect the quality of experimental data [1]. Endpoint data gathered from different literature sources or from different laboratories are more likely to reduce the predictive quality of the final model [37], even thought this is sometimes unavoidable in order to obtain well-populated datasets suitable for modeling. In such cases, it is important that the data are consistent and come from an analogous experimental protocol.

A common scenario in these cases is the presence of duplicates in different sources with different experimental values. If the difference among values is large, all the duplicates should be excluded because the difference probably reflect an error in one or more records. If differences are minimal, they may well be related to experimental variability. In this case, some simple options (e.g. average values or most conservative values) may be considered to aggregate values.

Another case is that both experimental properties are correct but the previous curation (for example, the removal of counterions in salts) modified the substance records to create such duplicates. For instance, the two records might correspond to two different salts of the same compound (or a neutral compound and its salt). As previously mentioned, the experimental properties can be very different if they are directly influenced by the counterion (e.g. large organic counterions). In some cases, even large differences could be explained by the fact that the experimental properties are reported in mg (e.g. endpoint expressed as mg/kg, mg/L) and the different molecular weight of counterions may be responsible for the different values. As stressed by Dearden et al. [8], the correct unit of measure when talking about doses or concentrations is the number of molecules (moles) and not the weight of the molecules (mg). Conversion of the unit from mg to mmol to account for differences in molecular weight between the two salts may therefor lead to closer experimental values. Some authors recognize the error introduced by neutralizing salts, because different salts or a salt and its neutral form may have different behaviours [11, 13]. They suggest excluding salts form the dataset as an alternative. Using the information reported in the output of the workflow, the user may also follow this option in salt treatment.

These simple basic treatments (quantification of the differences in activity between duplicates, aggregation of experimental values, and conversion of the endpoint unit to mmol) will be considered for implementation in future versions of the workflow. This technical improvement is not particularly difficult considering the flexibility of KNIME workflows and the possibility of easily modifying and adapting them to various needs.

## Conclusions

In the last few years, several publications have summarized the main good practices that should be applied in data curation [2, 11–14]. High-quality data is essential to obtain robust and predictive QSARs, and ignoring this will invalidate all the subsequent steps of model derivation. Despite this, to date only few attempts have been made to formalize and implement these good practices in an automated and usable tool. The present study meets the invitation made by Fourches et al. [13] which encourages experts to contribute their knowledge and best practices for dealing with the issues related to data curation. Following this invitation, the authors in turn encourage the expert community to apply this tool in their scientific work and improve the workflow based on their knowledge and experience. Our hope is that this tool will serve in future as a valuable support for researchers.

## Additional files

**Additional file 1.** Workflow's guideline for users.

**Additional file 2.** Output of the curation procedure for the Obach et al. [17] dataset.

**Additional file 3.** Output of the curation procedure for the SIN List [18].

**Additional file 4.** Output of the curation procedure for the EPISuite™ solubility dataset.

## Abbreviations

CIR: chemical identifier resolver
CAS: chemical abstracts service
CDK: chemistry development kit
QSAR: quantitative structure activity relationship
REST: representational state transfer
SMILES: simplified molecular input line entry system
