# Correction to: A new semi-automated workflow for chemical data retrieval and quality checking for modeling applications

**DOI:** 10.1186/s13321-019-0353-8

**Published:** 2019-04-25

**Authors:** Domenico Gadaleta, Anna Lombardo, Cosimo Toma, Emilio Benfenati

**Affiliations:** Laboratory of Environmental Chemistry and Toxicology, Department of Environmental Health Sciences, Istituto di Ricerche Farmacologiche Mario Negri IRCCS, Via la Masa 19, 20156 Milan, Italy

## Correction to: J Cheminform (2018) 10:60 10.1186/s13321-018-0315-6

It was highlighted that the original article [1] contained an error in the Funding section. This Correction article states the correct and incorrect versions of the Funding section.

**Correct Funding**

This work has received funding from the European Union’s Horizon 2020 research and innovation programme under Grant Agreement No. 681002 (EU-ToxRisk) and from the LIFE Project LIFE15 ENV/ES/000416 (LIFE-COMBASE).

**Incorrect Funding**

This study was funded by EU-ToxRisk (Project Reference: 681002) and LIFE-COMBASE (LIFE15 ENV/ES/000416).
