# Supplementary material for: A new semi-automated workflow for chemical data retrieval and quality checking for modeling applications
Source: J Cheminform. 2018 Dec 10;10:60. doi: 10.1186/s13321-018-0315-6 (PMC6503381; doi:10.1186/s13321-018-0315-6)
Supplement: Supplementary file 1 — Additional file 1. Workflow's guideline for users. [file 13321_2018_315_MOESM1_ESM.pdf]

A NEW SEMI-AUTOMATED WORKFLOW FOR CHEMICAL DATA RETRIEVAL AND QUALITY CHECKING FOR MODELLING APPLICATIONS

A semi-automated procedure is made available to support scientists in data preparation for modelling purposes. The procedure address:

1. **Automatic chemical data retrieval** (i.e., SMILES) from different, orthogonal web based databases, by using two different identifiers, i.e. chemical name and CAS registration number. Records were scored based on the coherence of information retrieved from different web sources.
2. **Data curation procedure** performed to top scored records. The procedure includes removal of inorganic and organometallic compounds and mixtures, neutralization of salts, removal of duplicates, checking of tautomeric forms.
3. **Standardization of chemical structures** yielding to ready-to-use data for the development of QSARs.

Installation

How to install the workflow

1. Install the last version of KNIME. It can be downloaded at https://www.knime.com/knime-analytics-platform (Windows version, 64bits).
2. Open KNIME.
3. Go to *"File -> Import KNIME Workflow"*.
4. Tick *"Select File:"* and go to *"Browse…"*. Select the *.knwf file of the workflow.
5. Click to *"Finish"*.

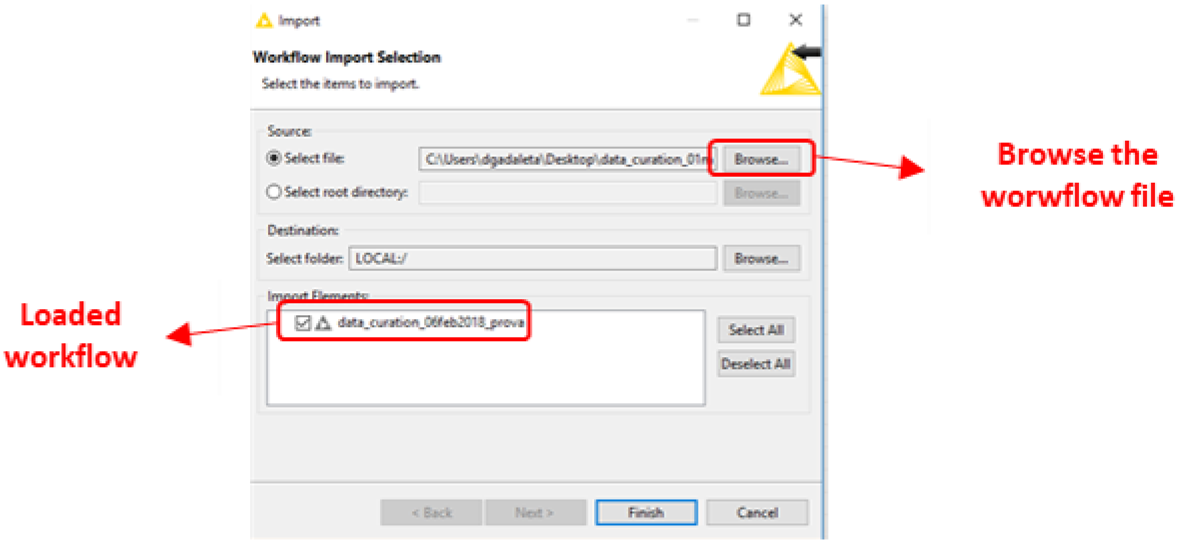

6. The data curation workflow now is in your "KNIME Explorer" menu on the left of the screen. Double click on the workflow to open it.

7. If some of the plugins used for the workflow are missing, a message will appear asking you to install the missing extensions. Click on *"Ok"*. The procedure will guide you in the installation of the missing extensions. Restart KNIME to make the new plugins working.

Use the Workflow

How to use the workflow – part 1

1. Prepare the input file. The file should be an Excel file (*.xlsx) with exactly three column. The columns can be in any order and should have an header:

- List of chemical names. Avoid the use of strange formatting (e.g. names on more lines).
- List of CAS numbers.
- List of IDs. IDs should be only numeric.

WARNING: Be sure that names and CAS numbers do not have any blank space at the end or at the beginning of the string. If some CAS numbers are missing in the list, do not leave the cell empty, but write something in the cell (e.g. NA).

2. Load the input file in the *"Select input"* wrapped metanode:

- Double click on the metanode.
- Click on *"Browse…"* and select the input file path.
- Clink on *"Ok"*.

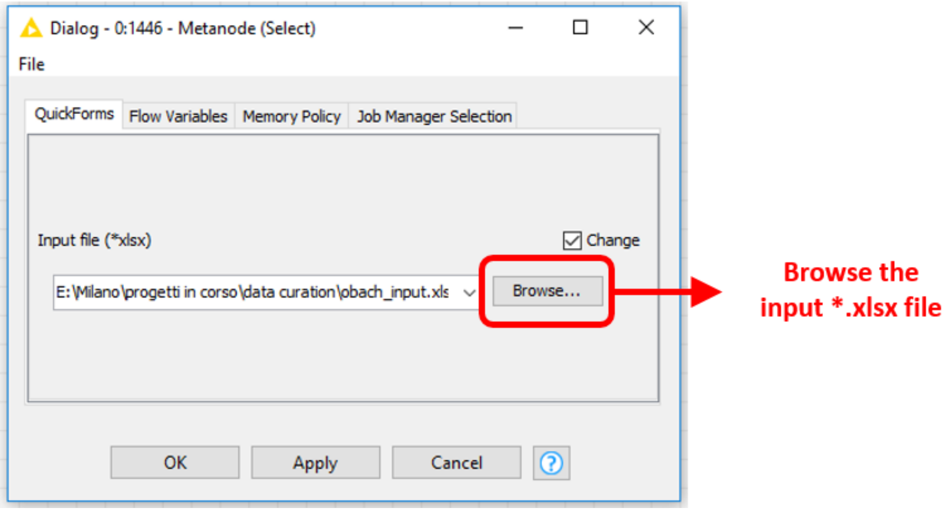

3. Execute the metanode by right clicking on it and then on " Execute" in the drop-down menu. If the input file has been correctly loaded, the "Select input" wrapped metanode will change from will turn from red to green . If the node return an error , some problems occurred during the loading of the input file.

4. Modify the settings in the second wrapped metanode, "Select columns and output"

- Double click on the metanode.
- Click on *"Browse…"* and select the path for the preliminar output file produced by the workflow. By default, the output file will be paced on the Desktop.

- Select the columns of the input file including the ID, the chemical names and the CAS numbers from the drop-down menus.
- Select the name of the preliminar output file. By default, the name of the output will be "out_db.xlsx".
- Clink on *"Ok"*.

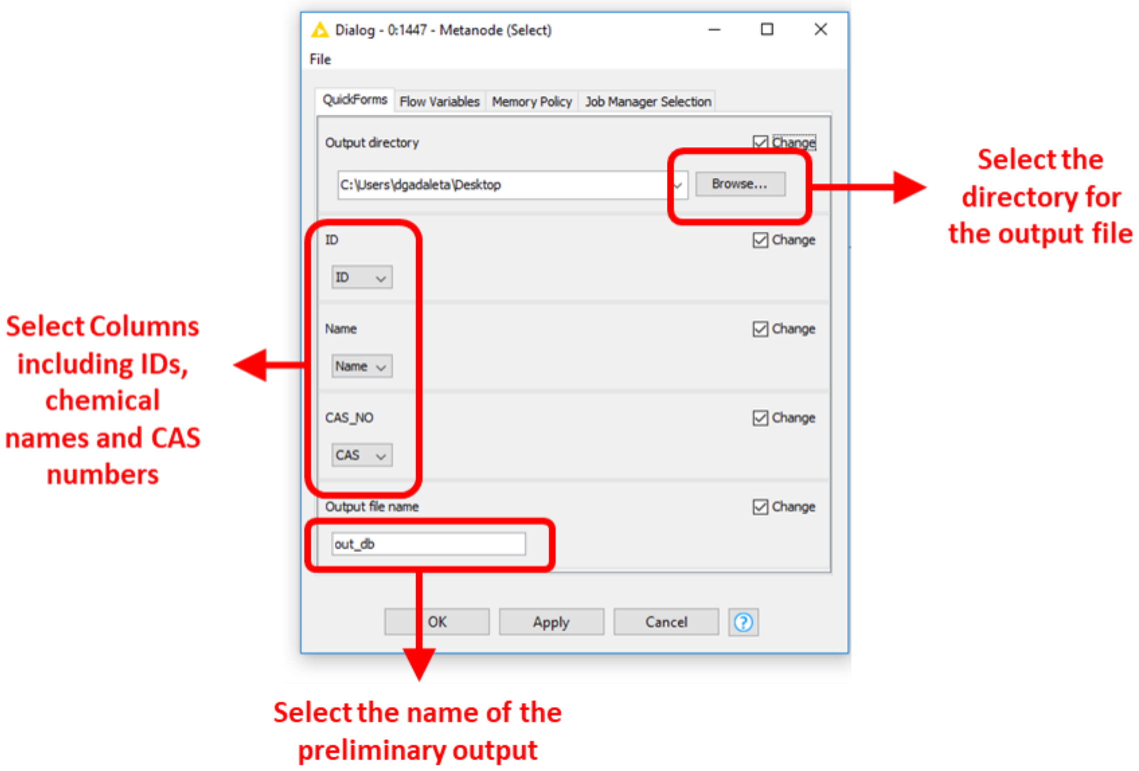

5. Execute the metanode by right clicking on it and then on " Execute" in the drop-down menu. If the input file has been correctly loaded, the "Select columns and output" wrapped metanode will change from will turn from red to the "running" state . If the first part of the workflow is successfully executed, the node will change to green . If the node give an error , some problems occurred during the loading of the input file.

6. Press on the top-left of the window to save the workflow.

WARNING: some time the workflow paused because it stops at the "Salts removal, neutralization and normalization" node that is inside the "Select columns and output -> Part I" node. You can check it by double clicking on the "Part I" node. When it happens, the "Salts removal, neutralization and normalization" node is paused while the nodes before are executed , but no nodes are running . In this case, try to execute again the workflow clicking on the url button some times, until the node restarts.

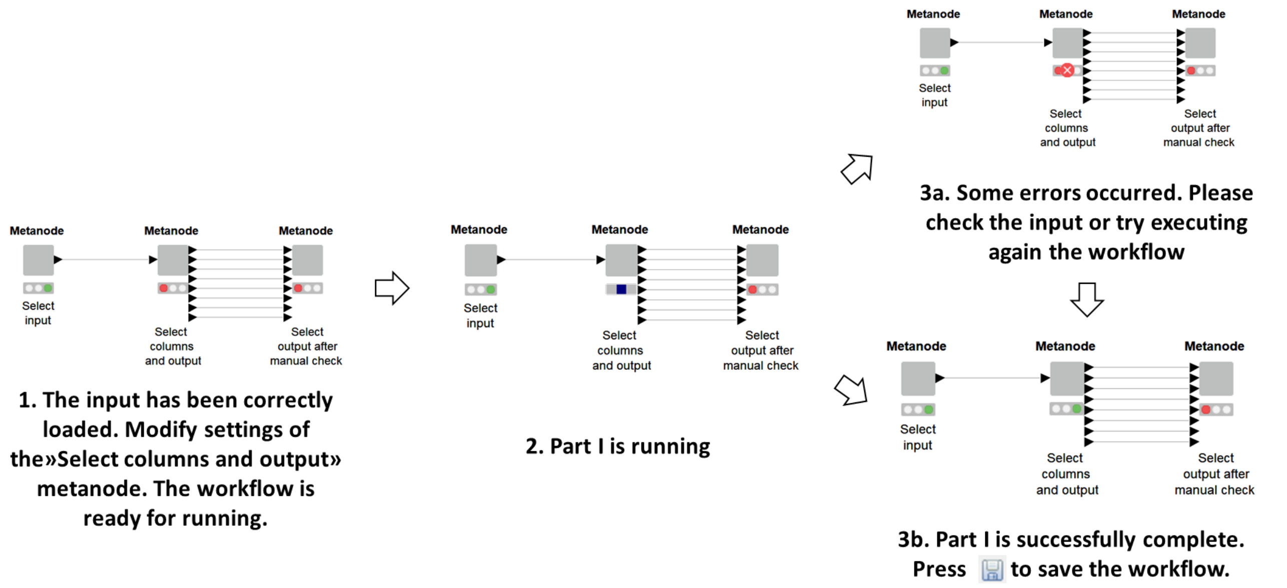

Manual check

1. If the first part of the procedure has been successfully completed, an output file (by default named "out_db.xlsx") will be produced on the previously indicated path (by default, it is on the Desktop). It include the various sheets with the following information:

- **Maintained**: list of compounds that passed the curation procedure and can be included in the final dataset. The sheet includes for each compound i) a new progressive ID for chemicals; ii) all the original SMILES retrieved from the web; iii) the common neutralized SMILES; iv) the SMILES normalized with VEGA; v) the number of duplicates for the record in the original dataset; vi) the list of names (with occurrences) assigned to the original records; vi) the list of CAS_NOs (with occurrences) assigned to the original records; vii) reliability (High or Medium); viii) list of possible warnings.
- **Rejected**: list of compounds that did not passed the curation procedure (e.g., inorganic compounds, mixtures, ambiguous chemicas). The sheet includes for each chemical i) ID; ii) name; iii) CAS_NO and iv) a collection of warnings indicating the cause of removal of the record.
- **Manual Check**: list of compounds that should be manually searched on the web. It includes i) ID, ii) Name, iii) CAS_NO, iv) SMILES collected from CIR and CompTox; v) number of Equal, Different and Missing SMILES; vi) a specification on the information that should be searched on the web (verify consistency of the name, search one or two further confirmations); vii) a list of synonyms retrieved from PubChem for a preliminary name verification.
- **Full Output**: it summarizing the information reported as above for all the chemicals.
- **Neutralized and Counterions**: it includes i) ID, ii) CAS_NO, iii) original SMILES, iv) neutralized SMILES and iv) counterions retrieved from CIR and CompTox using Name and CAS_NO as identifier; v) warnings on couterions.
- **PubChem_ChemID_check**: includes the same information as above that are results of the automated check made on PubChema and ChemID.
- **Summary**: it reports the number of Maintaineded (including and excluding duplicates, with details on reliability), web check and rejected chemicals.
- **Counterions_CompTox /CIR_CAS/Name**: four parts that report i) original SMILES; ii) SMILES stripped of counterions; iii) neutralized SMILES; iv) list of counterions and v) MW of molecules and counterions; vi) possible warnings for neutralization.

2. Open the output file and go to the *"Web Check sheet"*. Fill the last *"Web confirmation column"* with the information required in the *"Notes"* column:

- **Verify name:** the automatic procedure found coherent SMILES from the CAS number but the SMILES retrieved from the chemical name are not coherent or missing at all. This may be caused often by a typo in the chemical name. First, verify if the chemical name is included to the list of synonyms included in the *"Synonyms"* column. If you can find the name in the list, or a very similar name is present in the list (e.g., the only differences are due to typos, missing/additional blank spaces or use of different type of parenthesis), insert *"1"* in the corresponding *"Web confirmation"* cell. If you cannot find the name in the list, you should use the chemical name to search a confirmation on a web-based database of the SMILES retrieved from the CAS number (that is in the *"smiles_DSSTox_CAS – Neutralized"* and *"smiles_CIR_CAS – Neutralized"* columns). If the confirmation is found, insert *"1"* in the *"Web confirmation"*, otherwise type *"0"*.
- **Search at least one confirmation:** the automatic procedure found coherent, but not complete information (i.e., two out of four SMILES are equal, the others are missing). The user should search a confirmation of the SMILES indicated in the excel file from both the chemical name and the CAS number for a source or a database on the web. If at least one confirmation is found, insert *"1"* in the corresponding *"Web confirmation"* cell. Otherwise, insert *"0"*.
- **Search at least two confirmations:** the same as above, but two confirmation of the SMILES indicated in the excel file should be found. Insert the number of confirmations (i.e., 1, 2 or 0) in the corresponding *"Web confirmation"* cell.

3. Save the file after completingthe manual check.

WARNING: the web datasets used for the manual search should be different form dataset used within the KNIME workflow:

- *EPA's chemistry dashboard/DSSTox (https://comptox.epa.gov/dashboard)*
- *Cactus (https://cactus.nci.nih.gov/chemical/structure)*
- *ChemID Plus (https://chem.nlm.nih.gov/chemidplus/)*
- *PubChem (https://pubchem.ncbi.nlm.nih.gov/)*

In case of industrial chemicals, the user can use some chemical supplier's websites, e.g. Chemical Book (http://www.chemicalbook.com/), Molbase (http://www.molbase.com) or Sigma-Adritch (www.sigmaaldrich.com). In case of drug chemical, the user can search some specific drug dartabases e.g., Drugbank (www.drugbank.ca). Other specific databases can be used based on the chemical class.

WARNING: be sure that no columns are hidden in the Web Check sheet before saving it. This may lead to errors in reading the input of the following phases of the curation procedure.

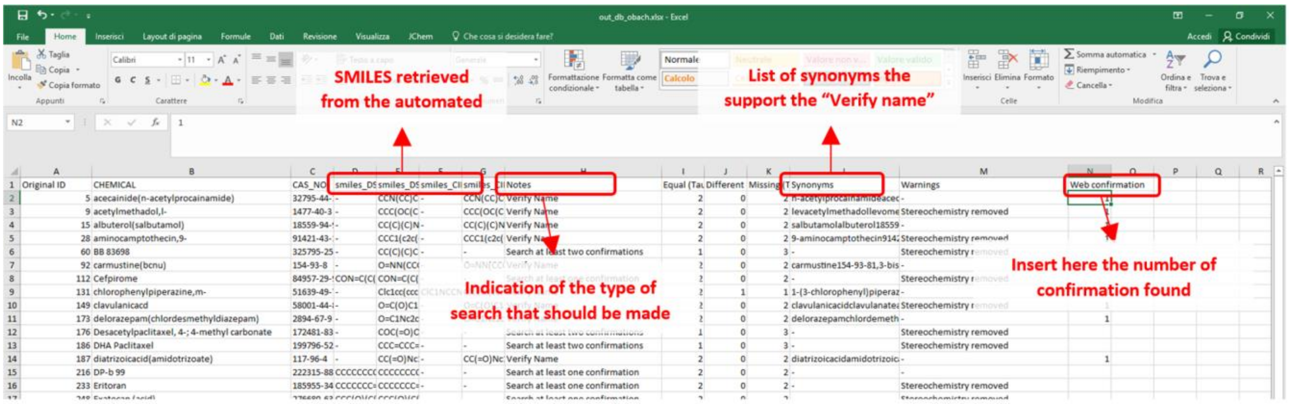

How to use the workflow – part 2

1. Modify the settings in the third wrapped metanode, "Select output after manual check"

- Double click on the metanode.
- Click on *"Browse…"* and select the path for the final output file produced by the workflow. By default, the output file will be placed on the Desktop.
- Select the name of the preliminar output file. By default, the name of the output will be the same of the preliminary output file. If the name final output is the same as the preliminary output, the excel file will be updated with new sheets.
- Clink on *"Ok"*.

2. Execute the "Select output after manual check" metanode by right clicking on it and then on " Execute" in the drop-down menu. The updated preiminary input file will be automatically loaded in the workflow. The "Select output after manual check" wrapped metanode will change from will turn from red to the "running" state . If the first final of the workflow is successfully executed, the node will change to green . If the node give an error , some problems occurred during the loading of the input file.
3. If the second part of the procedure has been successfully completed, the final output will appear on the indicated directory (by default, on the Desktop), or the preliminary output file will include new sheets:

- **Maintain_webcheck**: list of maintained chemicals updated with those that passed the manual check step. This is the final output of the curation procedure.
- **Rejected_webcheck**: list of rejected chemicals updated with those that did not passed the manual check. A *"web check failed"* flag is added to those records.
- **Full_output_webcheck**: it is the same as *"Full_output"* with the exception that Web Check chemicals are flagged as *"Maintain"* or *"Reject"* based on manual check results.
- **Summary_webcheck**: it updates the previous Summary with results of the manual curation.

WARNING: the workflow you use for the second part of the data curation procedure should be the same you saved after the first part. The "Select columns and output" metanode should be in the green state . If "Select columns and output" metanode is in the red or error state, then the first part of the procedure has been probably run on different data, and the second part of the procedure may return a wrong output.

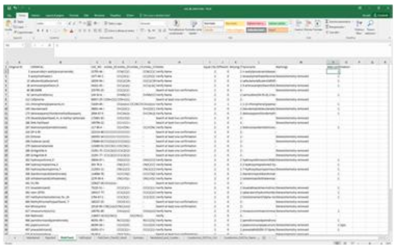

1. Open the «out_db.xlsx» file. Complete the manual check. Save the Excel file.

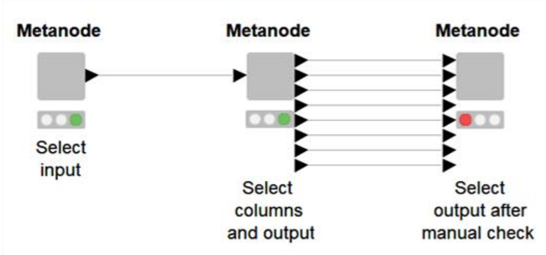

2. Return to the workflow. Modify settings of the «Select output after manual check» metanode. Press

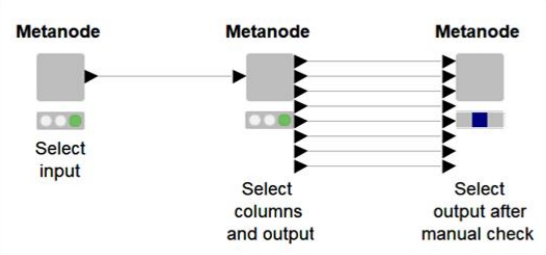

3. Part II is running…

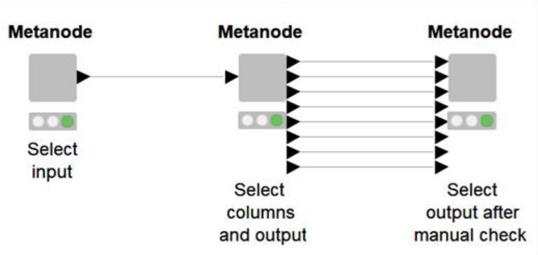

4. Part II is successfully complete. Press to save the workflow. Check the «out_db.xlsx» file for final results.

Tips and tricks

1. Be sure of visually inspect counterions of maintained chemicals. They may reveal some kind of chemicals that should be removed but that the procedure was not able to identify:

- An *"organic counterion"* flag may reveal the presence of mixtures. In this case, the chemical has one or more organic counterions that are equal to the main molecule.
- *"Organic counterion"+"Organometallic"* may reveal the presence of coordination complexes. In this case, the chemical has a metallic counterion and one or more organic counterions that are equal to the main molecule.
